# Place of the ^18^F-FDG-PET/CT in the Diagnostic Workup in Patients with Classical Fever of Unknown Origin (FUO)

**DOI:** 10.3390/jcm10173831

**Published:** 2021-08-26

**Authors:** Simon Letertre, Pierre Fesler, Laetitia Zerkowski, Marie-Christine Picot, Jean Ribstein, Philippe Guilpain, Vincent Le Moing, Denis Mariano-Goulart, Camille Roubille

**Affiliations:** 1 Department of Internal Medicine, Lapeyronie University Hospital, CHU Montpellier, University of Montpellier, 34090 Montpellier, France; s-letertre@chu-montpellier.fr (S.L.); p-fesler@chu-montpellier.fr (P.F.); l-zerkowski@chu-montpellier.fr (L.Z.); jean.ribstein@umontpellier.fr (J.R.); 2 PhyMedExp, INSERM U1046, CNRS UMR 9214, University of Montpellier, CEDEX 05, 34295 Montpellier, France; d-mariano_goulart@chu-montpellier.fr; 3 Centre d’Investigation Clinique 1411, Clinical Research and Epidemiology Unit, Department of Medical Information, INSERM, CHU Montpellier, University of Montpellier, 34090 Montpellier, France; mc-picot@chu-montpellier.fr; 4 Department of Internal Medicine, Saint-Eloi University Hospital, CHU Montpellier, University of Montpellier, 34090 Montpellier, France; p-guilpain@chu-montpellier.fr; 5 IRMB, INSERM, CHU Montpellier, University of Montpellier, 34090 Montpellier, France; 6 Department of Infectious Diseases, CHU Montpellier, University of Montpellier, 34090 Montpellier, France; v-le_moing@chu-montpellier.fr; 7 Department of Nuclear Medicine, CHU Montpellier, University of Montpellier, 34090 Montpellier, France

**Keywords:** fever of unknown origin, ^18^F-FDG-PET/CT

## Abstract

Objective: To explore the diagnostic contribution of the ^18^F-FDG-PET/CT in a population of patients with classical fever of unknown origin (FUO), to pinpoint its place in the diagnostic decision tree in a real-life setting, and to identify the factors associated with a diagnostic ^18^F-FDG-PET/CT. Method: All adult patients (aged ≥ 18 years) with a diagnosis of classical FUO who underwent an ^18^F-FDG-PET/CT in the University Hospital of Montpellier (France) between April 2012 and December 2017 were included. True positive ^18^F-FDG-PET/CT, which evidenced a specific disease causing FUO, were considered to be contributive. Results: Forty-four patients with FUO have been included (20 males, 24 females; mean age 57.5 ± 17.1 years). Diagnoses were obtained in 31 patients (70.5%), of whom 17 (38.6%) had non-infectious inflammatory diseases, 9 had infections (20.5%), and 3 had malignancies (6.8%). ^18^F-FDG-PET/CT was helpful for making a final diagnosis (true positive) in 43.6% of all patients. Sensitivity and specificity levels were 85% and 37%, respectively. A total of 135 investigations were performed before ^18^F-FDG-PET/CT, mostly CT scans (93.2%) and echocardiography (59.1%), and 108 after ^18^F-FDG-PET/CT, mostly biopsies (including the biopsy of a temporal artery) (25%) and MRIs (34%). In multivariate analysis, the hemoglobin level was significantly associated with a helpful ^18^F-FDG-PET/CT (*p* = 0.019, OR 0.41; 95% CI (0.20–0.87)), while the CRP level was not associated with a contributive ^18^F-FDG-PET/CT. Conclusion: ^18^F-FDG-PET/CT may be proposed as a routine initial non-invasive procedure in the diagnostic workup of FUO, especially in anemic patients who could be more likely to benefit from ^18^F-FDG-PET/CT.

## 1. Introduction

Fever of unknown origin (FUO) [1,2] is one of the most challenging diagnostic situations, with about 200 possible underlying diagnoses. Classical FUO refers to a prolonged febrile illness for at least 3 weeks, with a body temperature >38.3 °C on several occasions, and without an established etiology despite extended investigations in non-immunocompromised patients [3]. Causes of classical FUO can be stratified into four categories: noninfectious inflammatory diseases (NIID), infections, malignancies, and miscellaneous causes [4,5].

The diagnostic workup of FUO includes a search for potential diagnostic clues (PDCs) that could help to ascertain a specific diagnosis, based on a complete medical history and symptom-taking, a physical examination, and several investigations, including cultures and standard imaging [6]. However, between 10 and 50% of patients remain undiagnosed. The main difficulty lies in the fact that diagnostic workup should be based on limited investigations with the highest diagnostic yield, those that are non-invasive and cost-effective as far as possible. Particularly in patients without PDCs, ^18^F-fluorodeoxyglucose (FDG) positron-emission tomography/computed tomography (PET/CT) is now considered a promising technique in the management of patients with FUO [6]. Indeed, FDG accumulates in cells with a high rate of glycolysis, such as activated leukocytes, in inflammatory conditions. Although giving a lower performance for assessing the urinary tract, gastrointestinal tract, and myocardium, ^18^F-FDG-PET/CT has been demonstrated to be of great interest in managing FUO [6]. The helpfulness of ^18^F-FDG-PET/CT to identify the underlying cause of FUO was described in several studies, ranging from 38 to 75% [6,7,8]. However, these studies were heterogeneous, and the place and performance of ^18^F-FDG-PET/CT in the FUO diagnostic workup remain yet to be determined.

The present study aimed to explore the diagnostic contribution of the ^18^F-FDG-PET/CT in a population of patients with classical FUO, to pinpoint its place in the diagnostic decision tree in a real-life setting, and to identify those factors associated with a diagnostic ^18^F-FDG-PET/CT.

## 2. Patients and Methods

### 2.1. Study Population and Design

This retrospective study included all adult patients (age ≥ 18 years) with a diagnosis of classical FUO who underwent an ^18^F-FDG-PET/CT in the University Hospital of Montpellier (France) between April 2012 and December 2017.

We retrospectively screened for eligible patients from the medical files of the nuclear medicine department to avoid referral selection bias, using prespecified search terms for the target condition (e.g., “prolonged fever”, “long-term fever”, “fever of unknown origin”, “recurrent fever”). Inclusion criteria were a diagnosis of FUO [2], defined as a febrile illness of more than 3 weeks’ duration, with an established body temperature >38.3 °C (>101 °F), without a diagnosis after history-taking, complete physical examination, and classical biological and radiological investigations that were left to the discretion of the physician, in addition to an ^18^F-FDG-PET/CT examination during the diagnostic process. Importantly, the temperature had to be measured and recorded in the medical file, to confirm fever and avoid declarative data alone. A recurrent fever [9] was defined as repeated episodes of FUO with fever-free intervals of at least 2 weeks and the apparent remission of symptoms. The exclusion criteria were non-classical FUO, i.e., immunocompromised patients with neutropenia (leukocyte count < 1.0 × 10^9^/L and/or granulocyte count < 0.5 × 10^9^/L, known human immunodeficiency virus (HIV) infection, known hypogammaglobulinemia (Ig < 50% of the normal value), or the use of the equivalent of more than 10 mg of prednisone for at least 2 weeks, as well as nosocomial fever [2]. This study is registered (French Commission Nationale Informatique et Liberté (CNIL) number 216910). The protocol of this study was approved by the Institutional Review Board of Montpellier University Hospital (IRB-MTP_2021_04_202100784).

### 2.2. ^18^F-FDG-PET/CT Acquisitions

All patients were instructed to fast for at least 6 h before 18-FDG injection. Serum glucose levels were measured using the hexokinase method. Whole-body emission and transmission scans were acquired in the 3D mode, 60 min after the intravenous administration of 3.5 MBq/kg 18-FDG. Nondiagnostic-quality non-contrast-enhanced CT images were acquired before the acquisition of the PET data. ^18^F-FDG-PET/CT was not performed for patients with hyperglycemia >2 g/L, for whom clinicians proposed to postpone ^18^F-FDG-PET/CT acquisition until after normalization of their glucose level. Quantitative assessment was performed for each metabolic target lesion using the standard uptake value (SUVmax).

### 2.3. Outcomes and Variables

Demographic and clinical characteristics were assessed, including age, gender, past medical history, and several accompanying symptoms such as night sweats, unintentional weight loss, or any specific organic symptom that could potentially offer diagnostic clues. The C-reactive protein (CRP) level, hemoglobin, leukocyte count, lactate dehydrogenase (LDH), ferritin, fibrinogen and gammaglobulins levels were determined. The times between the beginning of fever and hospitalization, and between the beginning of fever and ^18^F-FDG-PET/CT acquisition, as well as the time between an ^18^F-FDG-PET/CT and final diagnosis, were also recorded. Moreover, in order to determine the sequential investigations that had been realized, before and after ^18^F-FDG-PET/CT, were reported for each patient, including CT scanning of the chest, abdomen and pelvis, magnetic resonance imaging (MRI), digestive endoscopy, bronchial endoscopy, bone scintigraphy, transthoracic or transoesophageal echocardiography, and invasive procedures such as a bone marrow biopsy, temporal artery biopsy, liver biopsy or other biopsies.

### 2.4. Final Diagnosis and ^18^F-FDG-PET/CT Helpfulness

As classically reported [9], diagnostic results were grouped by infectious diseases, non-infectious inflammatory diseases, malignancy, other diseases, or no diagnosis. To assess the helpfulness of ^18^F-FDG-PET/CT, results were stratified into four categories: true positive, true negative, false positive, and false negative. ^18^F-FDG-PET/CT was categorized as a true positive or helpful when it revealed a specific disease to be causing FUO that could be confirmed with a biopsy or be clinically ascertained. Thus, only a true positive ^18^F-FDG-PET/CT that led to a final diagnosis was considered helpful. ^18^F-FDG-PET/CT was categorized as a true negative when it was normal or with some aspecific tracer uptakes that were not contributory to any diagnosis, with no cause of FUO further identified despite extensive procedures. It was categorized as a false positive when the detected results were considered to be unrelated to the condition causing the FUO, and a false negative when the ^18^F-FDG-PET/CT was normal but a particular disease was further evidenced with other tests. The helpfulness of ^18^F-FDG-PET/CT was independently assessed according to these four categories by two different members of the study team (one senior and one fellow).

### 2.5. Statistical Analyses

Descriptive statistics are presented as mean ± SD or number (%) where appropriate. The Mann–Whitney U test (or Student’s t-test) was used to compare continuous variables and chi-square test (or Fisher’s exact test) to compare the categorical variables. The *p* values < 0.05 were considered significant, and all statistical tests were two-sided. Furthermore, we calculated the sensitivity and specificity (and their 95% confidence interval), as well as the positive predictive value (PPV) and negative predictive value (NPV) of the ^18^F-FDG-PET/CT. All statistical analyses involved the use of an SAS V7.12 (SAS Institute, Cary, NC, USA).

## 3. Results

### 3.1. Study Sample and Patients’ Characteristics

Based on the screening of 2021 ^18^F-FDG-PET/CT tests from the database of the nuclear medicine department between April 2012 and December 2017, according to the inclusion criteria we selected 72 patients who underwent ^18^F-FDG-PET/CT to explore an FUO. After excluding 28 patients who did not have a classical FUO (e.g., nosocomial FUO), or the workup was without any temperature measurement reported in the medical file, we analyzed the data from 44 patients.

**Figure 1:**
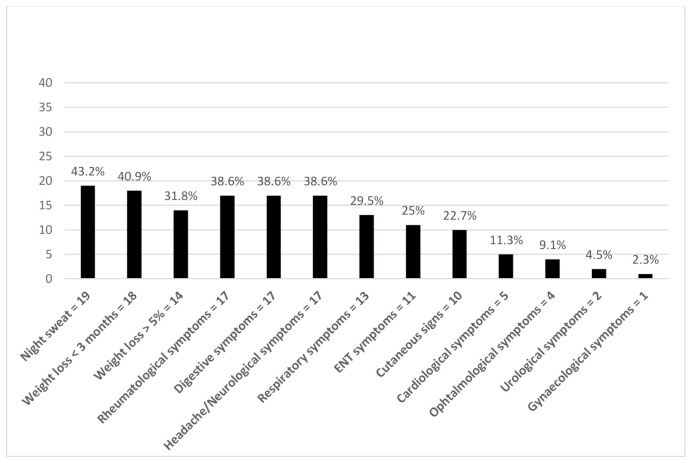
Clinical symptoms of the study population. (Abbreviation: ENT, ear, nose, and throat).

The study population comprised 44 patients (20 males, 24 females; mean age 57.5 ± 17.1 years), of whom 33 (75%) had a continuous FUO and 11 (25%) suffered from periodic FUO. Patients were referred by internal medicine departments (*n* = 16) or the department for infectious diseases (*n* = 25). Patients’ characteristics are summarized in Table 1. The most common, clinical symptoms reported by the patients were night sweats (43%) or recent weight loss (41%), mostly of > 5% of body weight (32%) (Figure 1).

### 3.2. Diagnosis

A diagnosis was obtained in 31 patients (70.5%), including 24 patients out of the 33 with continuous fever (72.7%) and 7 out of the 11 patients with periodic fever (63.6%). Out of these 31 patients, 17 (38.6%) had NIID, 9 patients had infections (20.5%), 3 patients had malignancies (6.8%) and 2 had other causes of fever (4.5%; chronic pericarditis and drug fever) (Table 2). In both continuous and periodic FUO, causes were mainly represented by NIID (39.4% and 36.4% respectively). The most frequent cause of FUO was large-vessel vasculitis (*n* = 8/17, of which 6 were giant cell arteritis). All the diagnoses are detailed in Table 3.

### 3.3. Position of the ^18^F-FDG-PET/CT in the Diagnostic Process 

**Figure 2:**
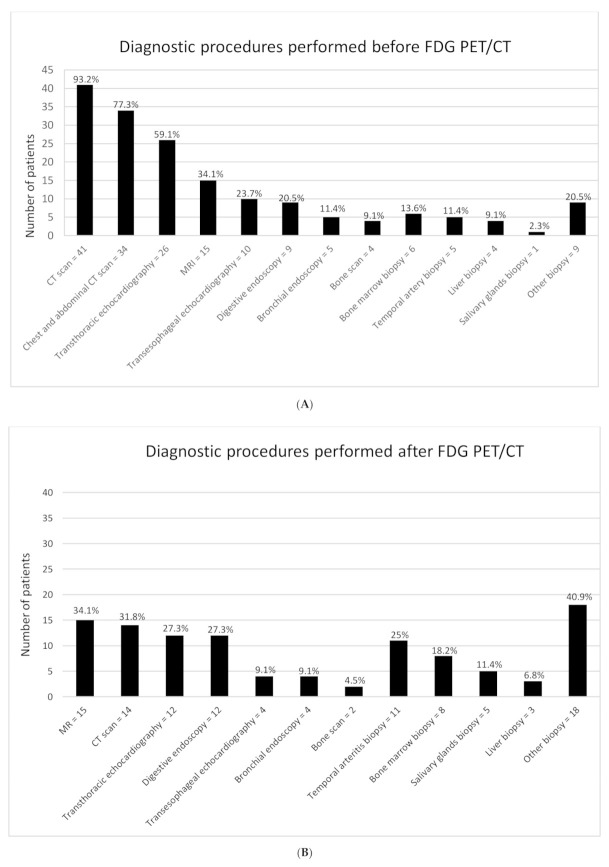
(**A**) Investigations that have been realized before ^18^F-FDG-PET/CT; (**B**) Investigations that have been realized after ^18^F-FDG-PET/CT.

The mean number of exams per patient was 5 ± 2.74. A total of 135 investigations of different kinds (e.g., CT scans, MRIs, echocardiography, endoscopy, a biopsy of the temporal artery, bone marrow biopsy, liver biopsy, or other biopsies) were performed before (Figure 2A) and 108 after ^18^F-FDG-PET/CT (Figure 2B). In addition, 47.7% of patients were referred to ^18^F-FDG-PET/CT after undergoing 2 exams, mostly CT scans (93.2%) and echocardiography (59.1%). After ^18^F-FDG-PET/CT, a mean of 2 investigations was performed in 59.9% of patients, mostly biopsy of the temporal artery (25%) or other biopsies, and MRI (34%).

### 3.4. Diagnostic Contribution of ^18^F-FDG-PET/CT

**Figure 3:**
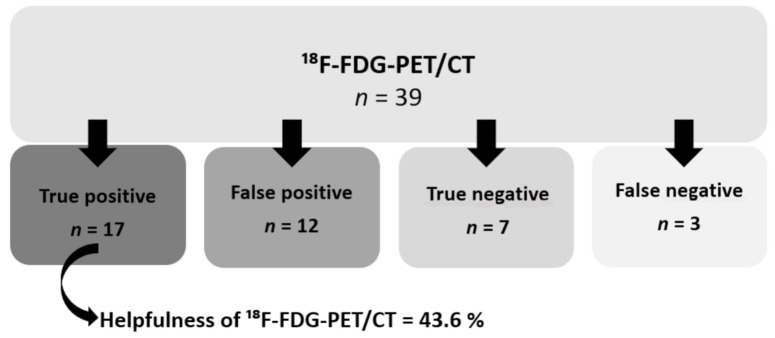
Diagnostic contribution of ^18^F-FDG-PET/CT.

Five patients were lost from follow-up after undergoing ^18^F-FDG-PET/CT without any diagnosis. Out of the 39 remaining patients, ^18^F-FDG-PET/CT was helpful for their final diagnosis (true positive) in 17 patients (43.6% of all patients and 54.8% of patients having a diagnosis) (Figure 3). Seven patients (17.9%) were found to have a true negative ^18^F-FDG-PET/CT, with no final diagnosis after workup. Twelve patients (30.8%) had a positive ^18^F-FDG-PET/CT with no relationship shown with the final diagnosis or with no final diagnosis (false positive). In 3 patients, we had false-negative findings (7.7%) while a final diagnosis could be made thanks to further investigations or in response to specific treatments. The sensitivity and specificity calculated were 0.85 (95% CI 0.69–1.00) and 0.37 (95% CI 0.15–0.58), respectively. PPV was 0.58 (95% CI 0.41–0.76) and NPV was 0.7 (95% CI 0.41–0.98). ^18^F-FDG-PET/CT showed a greater yield in continuous FUO (50%) compared to periodic FUO (22.2%) although this difference was not statistically significant (*p* = 0.25). Moreover, out of the 31 final diagnoses, ^18^F-FDG-PET/CT was contributive to the diagnoses of 41.2% of the NIID, 66.7% of the infectious diseases, and of all malignancies.

### 3.5. Diagnostic Contribution of Other Investigations in the Diagnostic Process

Out of the 31 patients with an FUO diagnosis, 11 chest and abdominal CT scans were found to be helpful for diagnosis, only one echocardiography (pericarditis), two MRIs (biliary tract infection), one bone CT scan (bone metastases), and one bronchial endoscopy (sarcoidosis). None of the 21 digestive endoscopies contributed to FUO diagnosis.

Twenty-eight biopsies were made before ^18^F-FDG-PET/CT, and 51 afterward (a total of 79 biopsies). Among those 51 biopsies performed in 31 patients (70.5%) after ^18^F-FDG-PET/CT, 18 biopsies (35%) were, among other things, based on ^18^F-FDG-PET/CT results, of which only 4 confirmed the final diagnosis. Among non-contributive biopsies, 42% were digestive biopsies. A temporal artery biopsy was performed in 16 patients (5 biopsies performed before and 11 performed after ^18^F-FDG-PET/CT, of which 4 were with the ^18^F-FDG-PET/CT suggesting vasculitis) but the scan was useful to confirm giant-cell arteritis in only 2 patients (12.5%), in one of whom ^18^F-FDG-PET/CT revealed large vessel arteritis. Only one liver biopsy of the 7 that were performed contributed to the final diagnosis (lymphoma). The same results were found for bone marrow biopsies, with only one positive contributive biopsy out of 14 (systemic mastocytosis). Regarding the lymph node biopsies, 10 were performed in 8 patients, of which 8 biopsies were guided by the results of ^18^F-FDG-PET/CT, leading to 3 diagnoses (2 of tuberculosis and 1 of sarcoidosis).

### 3.6. Predictors of High-Yield ^18^F-FDG-PET/CT

The clinical and biological characteristics of patients with an ^18^F-FDG-PET/CT that was helpful for diagnosis were compared to those with an ^18^F-FDG-PET/CT that was not contributive. In the univariate analysis (Table 4), the absence of neurological (*p* = 0.02) or cutaneous (*p* = 0.01) symptoms, as well as anemia (*p* = 0.01), were associated with the diagnostic accuracy of ^18^F-FDG-PET/CT. The latter result was confirmed in multivariate analysis with hemoglobin level significantly associated with helpful ^18^F-FDG-PET/CT (*p* = 0.019, OR 0.41 95%CI (0.20–0.87)). In the multivariate analysis, the absence of neurological (*p* = 0.018, OR 0.09, 95%CI (0.01–0.66)) or ENT symptoms (*p* = 0.014, OR 0.03, 95% CI (0.00–0.50)) were also associated with a helpful ^18^F-FDG-PET/CT. The CRP level was not associated with contributive ^18^F-FDG-PET/CT (*p* = 0.50).

## 4. Discussion

FUO is still a condition that is challenging to diagnose in the field of internal medicine, with the inherent risk of missing any serious disease. The spectrum of diseases causing FUO is large and has changed over the last twenty years, with NIID being more prevalent than infectious conditions, especially in Western countries [10]. Nevertheless, between 10 and 50% of FUO remain undiagnosed [11,12]. In our population, a final diagnosis could be reached in 70.5% of patients. Regarding the underlying diseases causing FUO, NIID were involved in 38.6% of cases, infections in 18.2%, malignancies in 9.1%, and miscellaneous diseases in 6.1%. Our results are in accordance with other studies [11,13,14], e.g., Zenone et al., who have reported that 74% of 144 patients received a diagnosis of FUO, of which 35.5% were of NIID, 30.8% of infectious diseases, and 13.5% of malignancies [15]. Among NIID, giant-cell arteritis was the most prevalent cause of FUO (18.2%), before adult-onset Still’s disease (6.8%) as in other studies [10,15,16]. We found a sensitivity of 85%, respectively, which is consistent with previous studies. Indeed, two meta-analyses, the first including 42 studies and the second including 23 studies, reported a pooled sensitivity of ^18^F-FDG PET/CT of 86% and 84%, respectively [17,18].

Until now, there was still no standardized diagnostic algorithm that is recommended in FUO. However, there is now some strong evidence that ^18^F-FDG-PET/CT may offer a great contribution to the diagnostic workup in FUO, with the helpfulness of ^18^F-FDG-PET/CT shown in 38–75% of patients [6]. In our present study, based on rigorous inclusion criteria and the definition of FUO, we found that ^18^F-FDG-PET/CT contributed to the final diagnosis in 43.6% of all patients and 54.8% of patients already having a diagnosis, with a sensitivity of 85% and a specificity of 37%. Our results are consistent with those in previous reports. In one prospective study including 48 patients with FUO, ^18^F-FDG-PET/CT showed a diagnostic contribution of 46% [19]. In one of the largest retrospective studies exploring the diagnostic performance of ^18^F-FDG-PET/CT, Gafter-Gvili et al. reported that ^18^F-FDG-PET/CT could lead to a final diagnosis in 46% of 112 patients with FUO [20]. In their meta-analysis, Bharucha et al. found a global helpfulness of ^18^F-FDG-PET/CT of 56% [7]. However, comparing the retrospective studies that have been included seems to be difficult given their heterogeneity, with some studies lacking FUO definition or with a different definition of FUO, others including immunocompromised patients. One prospective study of 240 patients has addressed the diagnostic contribution of ^18^F-FDG-PET/CT in both FUO (*n* = 72) and patients with inflammatory syndrome of unknown origin (IUO), some of whom had no fever [8]. Of all 190 patients with a final diagnosis (79.2%), ^18^F-FDG-PET/CT returned a true positive in 136 patients (71.6%; hence, 56.7% of all patients). Moreover, while only 5% were found to be a false negative, 30% were false positive. These findings demonstrated a sensitivity of 91.8% and specificity of 21.7%. Nonetheless, this study did not differentiate FUO from IUO, so that it seems difficult to compare these findings to ours. One remaining concern lies in the definition of a helpful ^18^F-FDG-PET/CT [21]. In our study, as in many others [6,21], we considered ^18^F-FDG-PET/CT helpful when it was a true positive and directly led to the final diagnosis of FUO. However, one may argue that a true negative ^18^F-FDG-PET/CT may also be considered clinically helpful for FUO diagnosis when no cause of FUO was further identified [21].

One strength of our study is to describe the position of ^18^F-FDG-PET/CT in the diagnostic workup process in a real-life clinical setting. Almost 95% of patients underwent a CT scan before ^18^F-FDG-PET/CT, mostly a chest and abdomen CT scan (77.3%). Interestingly, the advantages of ^18^F-FDG-PET/CT are the non-influence of results by metallic implants, whole-body screening, and the absence of contrast-related side-effects, while having the anatomical input of an integrated CT scan [22]. Moreover, gastroscopy and colonoscopy were never useful, as in Bleeker-Rovers et al.’s study [4]. Histological investigations were useful when guided by ^18^F-FDG-PET/CT, especially the temporal artery biopsy and lymph node biopsy, while liver and bone marrow biopsies were frequently non-contributive. All these findings are consistent with those of Bleeker-Rovers et al. [4]. Our data, thus, suggest that ^18^F-FDG-PET/CT should be performed quite early in the workup of FUO, after echocardiography, if deemed necessary, and in place of a thoracoabdominal CT scan. Histological biopsies might be performed only when guided by anomalies shown by ^18^F-FDG-PET/CT. The indication for digestive endoscopic studies should be carefully weighed.

As FUO is a very heterogeneous condition, identifying subgroups of patients with a higher chance of benefiting from ^18^F-FDG-PET/CT is of interest in FUO diagnostic development. In this context, finding that anemia was significantly associated with helpful ^18^F-FDG-PET/CT (*p* = 0.019) is of great interest. Similarly, Crouzet et al. showed that anemia is significantly predictive of ^18^F-FDG-PET/CT’s contribution in the FUO diagnostic [23]. Low hemoglobin may be considered a marker of inflammation. However, we did not find any association between CRP and the helpfulness of ^18^F-FDG-PET/CT. This association remains controversial, some studies reporting a significant association between CRP thresholds and ^18^F-FDG-PET/CT contribution [8,22,23], while others did not [16,24,25]. In their prospective FUO/IUO study, Shonau et al. found that ^18^F-FDG-PET/CT showed a better performance in patients older than 50 years, or those with CRP > 30 mg/L and no fever, and proposed that ^18^F-FDG-PET/CT could be performed earlier in these patients [8]. Further studies seem necessary to explore this relationship between CRP levels and the helpfulness of ^18^F-FDG-PET/CT in FUO.

Our study has several limitations. First, it has the inherent limitations of a retrospective observational study, with inclusion bias. Secondly, some patients have been treated with antibiotics or corticosteroids (<2 weeks), which may have reduced the ^18^F-FDG-PET/CT helpfulness. Nevertheless, this point reflects the therapeutic process in real-life conditions. Third, the clinical performance of the referring physicians, and the entire diagnostic process prior to ^18^F-FDG-PET/CT, could also have created a bias in patient selection. Finally, the size of the study population might imply relatively low power. However, this size is consistent with most of the published retrospective studies.

## 5. Conclusions

In summary, the present data argue for an ^18^F-FDG-PET/CT-based diagnosis of FUO. We showed that ^18^F-FDG-PET/CT allowed eventual diagnosis in 43.6% of patients. Therefore, ^18^F-FDG-PET/CT may be proposed as a routine initial non-invasive procedure in the diagnostic workup of FUO, especially in anemic patients who could be more likely to benefit from a helpful ^18^F-FDG-PET/CT.

## Figures and Tables

**Table 1:** Demographic, clinical, and biological characteristics of the study population.

	FUO Population (*n* = 44)
Age (years), mean (SD)	57.5 ± 17.1
Male, *n* (%)	20 (45.5%)
Medical history, *n* (%)	
NIID	7 (15.9%)
Malignancies	6 (13.6%)
Infectious diseases	5 (11.4%)
Diabetes	4 (9.1%)
Antibiotics use, *n* (%)	19 (43.2%)
Corticosteroids use, *n* (%)	5 (11.4%)
Continuous fever, *n* (%)	33 (75%)
Periodic fever, *n* (%)	11 (25%)
Referral departments, *n* (%)	
Infectious diseases center	25 (56.8%)
Department of Internal Medicine	16 (36.4%)
Other	3 (6.8%)
Year of realization of ^18^F-FDG-PET/CT, *n* (%)	
2012	4 (9.1%)
2013	7 (15.9%)
2014	9 (20.5%)
2015	10 (22.7%)
2016	10 (22.7%)
2017	4 (9.1%)
Time between the beginning of fever and hospital care, days, median (min-max)	22 (0–2300)
Time between the beginning of fever and ^18^F-FDG-PET/CT, days, median (min-max)	63 (22–7300)
Length of hospital stay before ^18^F-FDG-PET/CT, days, median (min-max)	13 (0–89)
Patients having PDCs, *n* (%)	40 (90.9%)
CRP, median (min-max) mg/l	72.2 (2.6–288)
Hemoglobin, median (min-max) g/dl	11.2 (8.1–14.6)
Leukocyte count, median (min-max) g/l	8.2 (3.2–31.2)

Abbreviations: CRP: C-reactive protein; FUO, fever of unknown origin; NIID: non-infectious inflammatory diseases; PDCs: potentially diagnostic clues.

**Table 2:** Diagnostic groups in patients with a final diagnosis among the study FUO population (*n* = 44).

	Final Diagnosis	NIID	Infection	Malignancy	Miscellaneous Disease
Continuous fever, *n* (%)	24 (72.7%)	13 (39.4%)	6 (18.2%)	3 (9.1%)	2 (6.1%)
Periodic fever, *n* (%)	7 (63.6%)	4 (36.4%)	3 (27.3%)	0	0
Total, *n* (%)	31 (70.5%)	17 (38.6%)	9 (20.5%)	3 (6.8%)	2 (4.5%)

Abbreviation: FUO, fever of unknown origin; NIID: non-infectious inflammatory diseases.

**Table 3:** Diagnoses obtained in FUO patients.

Diagnosis	Number of Patients
NIID	**17**
Giant cell arteritis	6
Takayasu arteritis	1
Large vessel vasculitis unclassified	1
Adult-onset Still’s disease	3
Sarcoidosis	1
Polymyalgia rheumatica	1
Antisynthetase syndrome	1
Aseptic abscesses syndrome	1
Chondrocalcinosis	1
Auto-inflammatory disease unclassified	1
Infectious disease	**9**
Nodal tuberculosis	2
Recurrent biliary tract infection	2
Actinomyces salpingitis	1
EBV meningitis with radiculitis	1
Vascular prosthesis infection	1
Pleuropneumonia	1
Bartonella Henselae endocarditis	1
Malignancy	**3**
Diffuse large B-cell lymphoma	1
Paraneoplastic fever (prostate cancer)	1
Systemic mastocytosis	1
Miscellaneous	**2**
Pericarditis	1
Drug fever	1

Abbreviation: FUO, fever of unknown origin; NIID: non-infectious inflammatory diseases; EBV: Epstein-Barr virus.

**Table 4:** Predictors of high-yield ^18^F-FDG-PET/CT (univariate analysis).

Outcome		Non-Contributive ^18^F-FDG-PET/CT	Contributive ^18^F-FDG-PET/CT (True Positive)	*p*-Value
Weight loss < 3 months	No	14	8	0.30
	Yes	8	9	
Weight loss > 5%	No	16	10	0.36
	Yes	6	7	
Night sweats	No	13	7	0.27
	Yes	9	10	
Rheumatologic symptoms	No	14	12	0.65
	Yes	8	5	
Digestive symptoms	No	14	11	0.94
	Yes	8	6	
Neurological symptoms	No	10	14	0.02
	Yes	12	3	
Respiratory symptoms	No	17	13	1.00
	Yes	5	4	
ENT symptoms	No	14	16	0.051
	Yes	8	1	
Cardiological symptoms	No	20	15	1.00
	Yes	2	2	
Cutaneous symptoms	No	15	17	0.01
	Yes	7	0	
Number of exams	Mean (SD)	3 (2.2)	2.7 (1.9)	0.76
CRP (mg/L)	Mean (SD)	97.3 (81.2)	78.7 (68.5)	0.50
Hemoglobin (g/L)	Mean (SD)	11.9 (1.3)	10.7 (1.4)	0.01

Abbreviations: CRP: C-reactive protein; ENT, ear, nose, and throat.

## Data Availability

Data could be available on reasonable request.
